# Role of transcription factors in apoptotic cells clearance

**DOI:** 10.3389/fcell.2023.1110225

**Published:** 2023-01-19

**Authors:** Yuqiong Gao, Yating Jiao, Xiaoyi Gong, Jie Liu, Hui Xiao, Qian Zheng

**Affiliations:** Key Laboratory of the Ministry of Education for Medicinal Plant Resources and Natural Pharmaceutical Chemistry, National Engineering Laboratory for Resource Development of Endangered Crude Drugs in the Northwest of China, College of Life Sciences, Shaanxi Normal University, Xi’an, China

**Keywords:** transcription factor, apoptosis, clearance of apoptotic cells, regulatory mechanisms, signal pathway

## Abstract

The human body generates 10–100 billion cells every day, and the same number of cells die to maintain homeostasis. The genetically controlled, autonomously ordered cell death mainly proceeds by apoptosis. Apoptosis is an important way of programmed cell death in multicellular organisms, timely and effective elimination of apoptotic cells plays a key role in the growth and development of organisms and the maintenance of homeostasis. During the clearance of apoptotic cells, transcription factors bind to specific target promoters and act as activators or repressors to regulate multiple genes expression, how transcription factors regulate apoptosis is an important and poorly understood aspect of normal development. This paper summarizes the regulatory mechanisms of transcription factors in the clearance of apoptotic cells to date.

## 1 Introduction

In multicellular organisms, billions of cells are fated to be dead and cleared away for the normal development, and the excessive or harmful cells undergo a programmed progress which is activated and regulated by a series of genes (Fuchs and Steller, 2011). Programmed cell death (PCD) is a highly evolutionarily conserved process, which involves in embryonic development, maintenance of homeostasis and immune system development (Jacobson et al., 1997). At present, apoptosis is one of the typical types of PCD, which is regulated by mutiple molecular mechanisms and results in tissue remodeling or killing pathogens (Toda et al., 2015). This review summarizes the underlying mechanisms of the transcriptional control in apoptosis or apoptotic cell clearance.

## 2 Apoptosis

Apoptosis is an important cell biological process in animal development, which is considered be essensial for organogenesis and maintaining daily tissue homeostasis. Once the apoptosis program is initiated, the signals released from ACs rapidly recruit and be captured by the professional phagocytes or neighboring cells, ultimately promoting the engulfment and elimination of cell corpses (Elliott and Ravichandran, 2016). Generally, the occurrence of apoptosis involves three main steps: the activation and transduction of apoptotic signals, the initiation of apoptotic program, finally the clearance of ACs. Apoptosis occurs with a range of biochemical and morphological characteristics changes, like membrane shrinkage, DNA disassembly, production of apoptotic bodies and engulfment by phagocytes. The pronounced expression of cell surface apoptotic markers initiates early migration and recognition, triggering timely and efficient phagocytosis to avoid damaging neighboring tissues.

The original study of apoptosis molecular mechanism is started by the accurate description of the cell lineage during ontogenesis of *Caenorhabditis elegans*, in which there are 1090 somatic cells produced and 131 cells dead through apoptosis procedure. Observation on mutants which affected the 131 somatic cell deaths during worm development discovered a series of specific genes controlled the apoptosis process, which were proved to exist and play important roles as a similar manner in mammals (Sulston and Horvitz, 1977). In cells fated to die, EGL-1 binds to CED-9 and directly inhibits the interaction between CED-9 and CED-4, resulting in translocation of CED-4 from the mitochondrial surface to the perinucleus, thus promotes the activation of the CED-3 caspase and cell death (15) (Conradt and Horvitz, 1998).

Since the researchers have proved the apoptosis process is complicated yet evolutionarily conserved from invertebrates to mammals, which is mainly mediated by two independent and classical pathways: the death receptor (DR) extrinsic apoptotic pathway and the mitochondria-triggered endogenous pathway (Wang and Su, 2018; Xie et al., 2018). The extrinsic pathway is initiated by DRs members of the tumor necrosis factor-α (TNF-α) receptor superfamily, which bind to the exogenous ligands through their extracellular cyteine-rich domains, and transduct the apoptosis signal with the cytoplasmic domain called “death domain”. Generally, the death domains are essential for the downstream activation of apoptosis signal, which have high homology among different DRs. Current well-researched DR, Fas forms a tripo lymer in the cell membrane to recruit the adaptor protein FADD and proenzyme caspase 8, producing a protein complex called death inducing signaling complex (DISC) to initiate an apoptotic cascade (Guicciardi and Gores, 2009). In the intrinsic signaling pathway of apoptosis, mitochondria plays an critical role, and the release of cytochrome c is the key event mediated by the Bcl-2 family, in which process alters the permeability of cell membrane (Martinou and Youle, 2011). The two apoptotic pathways above seem to have totally different regulatory mechanisms, but they are both caspase dependent, where the caspases are cleavaged and activated to initiate the apoptosis through the amplified caspase cascade (Elmore, 2007; Abdolmaleki et al., 2018).

## 3 Apoptotic cell clearance

ACs that undergo programmed cell death are usually eugulfed by “professional” phagocytes or other cells timely and effectively, to avoid inflammatory responses or maintain homeostasis. Failure in this process would cause redundant ACs accumulation in the organ, ultimately lead to the development of inflammatory autoimmune or neurodegenerative diseases. Therefore, timely and effective clearance of apoptotic cells in multicellular organisms is of great importance for the homeostasis of the organism (Doran et al., 2020).

Before being recognized by phagocytes, ACs release or expose some different signal molecules, which may in turn promote the recognition and elimination of ACs. The process of clearing ACs by macrophages can be generally divided into four processes: recruitment, recognition, phagocytosis, and degradation (Wang and Yang, 2016). Through the study of model organisms such as *C.elegans*, *Drosophila melanogaster*, researchers have uncovered the molecular mechanism of ACs clearance by phagocytes. Differently, mammalian macrophages are divided into two sub groups according to their functions and levels of inflammatory cytokines (Roszer, 2015). The M1 macrophages (classically activated macrophages) are mainly activated by lipopolysaccharide produced by bacteria and cytokines, promoting to kill bacteria or inflammation (Rőszer, 2015). During clearance of ACs, macrophages are influenced by several factors (such as IL4) and polarize towards the M2 state. While M2 activation is involved in apoptotic cell clearance, wound healing is promoted and inflammation is inhibited (Lawrence and Natoli, 2011).

Apoptosis is also a ubiquitous process during epithelial morphogenesis and homeostasis, and typical mechanisms to maintain homeostasis include apical extrusion of apoptotic cells and phagocytes removal of residual cellular debris (Rosenblatt et al., 2001; Grieve and Rabouille, 2014). Epithelial cells and fibroblasts, as two typical non-professional macrophages, can absorb a limited range of apoptotic particles and play a certain degree of clearance (Rabinovitch, 1995). In recent years, such clearance mechanisms have been observed in the epithelial tissue areas of the lungs, thymus, and breasts (Cao et al., 2004; Monks et al., 2005; Juncadella et al., 2013). Unlike typical professional macrophages, epithelial cells perceive and process their environment more often from changes in actin and cadherin signals, rather than from typical transcription factors: the actin regulatory factor Coronin 1B promotes the formation of integrated actin networks through adhesion and recruitment of E-cadherin during biogenesis at the cell-cell junction to help it achieve optimal contraction and cyclic ability (Lubkov and Bar-Sagi, 2014; Michael et al., 2016).

### 3.1 Recruitment

When the process of apoptosis occurs, the ACs will generate and release some chemokines or other signals to recruit phagocytic cells. Meanwhile, in mammals, in addition to releasing find me to attract certain phagocytic cells, ACs also release “keep out” signaling factors to block the approach of inflammatory cells such as neutrophils. Lactoferrin, a multifunctional glycoprotein, is the only protein identified to date that acts as a “keep out” signal. “Find me” signalincludes lysophosphatidylcholine (LPC) (Lauber et al., 2003) and sphingosine 1-phosphate (S1P), nucleotides (including ATP and UTP 16), and chemokine CX3CL1 (fractalkine), etc. It has been shown that H_2_O_2_ may act as a “find me” signal in *Drosophila* embryos, which is required for the recruitment of blood cells to the wound area (Moreira et al., 2010). During the H_2_O_2_-induced wounding response, Src42A-Draper-Shark signaling was found to be essential for the hemocyte recruitment (Evans et al., 2015).

### 3.2 Identification

The cellular environment is very complex, including healthy cells, ACs, lymphocytes, and phagocytic cells. Therefore, the recognition of ACs by phagocytes is very crucial, and this process mainly depends on the molecules exposed to the apoptotic cell surface such as phosphatidylserine, Intercellular adhesionmolecule 3 (ICAM3) (Kristóf et al., 2013), Calreticulin (Gardai et al., 2005), oxidized low-density lipoprotein (Di and Maiseyeu, 2021), glycosylated surface proteins, etc. These signal molecules can be usually perceived as “eat me” signals, which distinguish ACs from healthy cells are easily recognized by phagocytes.

The most well-known and highly conserved “eat me” signal is phosphatidylserine (PS), phagocytes directly or indirectly recognize and bind these signals through their own PS recognizing membrane receptor (PSR), thereby promoting the recognition and engulfment during apoptosis and preventing inflammation. The trigger receptor and advanced glycation end product receptor of Brain Angiogenesis Inhibitor 1/3(BAI1/3), T cell immunoglobulin mucin receptor 4 (TIM4), Stabilin-1/2 (Stab2), (TREM)-like protein 2 (TLT2) are identified as directly binding to PS (Miyanishi et al., 2007; Park et al., 2007; Park et al., 2008; He et al., 2011). The recognition between PS and phagocytic receptors can also be mediated by bridging molecules, such as phagocytic receptor α _v_ β_3_ integrin binds to ACs through PS-dependent bridging molecule MFG-E8; TAM receptor (receptor tyrosine kinase Mer Tyro3 and Axl, known as TAM receptors) recognizes PS through interacting with growth arrest-specific gene 6 (Gas6) or protein S (Savill et al., 1990; Nakano et al., 1997; Scott et al., 2001; Hanayama et al., 2002). Other bridging molecules including C1q, MBL, TSP-1 and TTR52 bind to phagocytic receptors LRP1, CRT, CD36 and MEGF10, respectively, in order to recognize PS (Savill et al., 1992; Ogden et al., 2001; Fond and Ravichandran, 2016). These receptors facilitate individually or coordinate with bridging molecules to promote the recognition of ACs.

We found that receptors on the surface of phagocytes are also highly conserved, such as in *Drosophila* Draper (Drpr, MEGF10 in mammals, CED-1 in worms), integrin and Croquemort (Crq, CD36 in mammals) and other phagocytosis receptors, which were found to function in different ways during phagocytosis: BAI1 is essential for phagosome formation, while TIM-4 stabilizes phagosomes (Mazaheri et al., 2014). In addition, healthy cells have a “Don’t eat me” signal that acts as an inhibitor to prevent phagocytosis by phagocytes, such as CD31, CD46 and CD47 (Poon et al., 2014).

Then macrophages integrate various signals from ACs and transmit them to the downstream, promoting a series of processes to recognize and engulf ACs.

### 3.3 Engulfment

Macrophages integrate signals from ACs to promote cytoskeletal rearrangement, a process that has been studied in the model organisms *C. elegans* and *Drosophila*, as well as in *mammals*. In *C. elegans*, upstream signals were found to converge in two parallel and independent signaling pathways:CED-2, CED-5, CED-12 pathway and CED-1, CED-6, CED-7 pathway which both subsequently activate CED-10, an evolutionarily highly conserved GTPase (Park and Kim, 2017) and thus stimulates skeletal rearrangement to form phagocytic vesicles (Wu and Horvitz, 1998a; Wu and Horvitz, 1998b; Liu and Hengartner, 1998; Reddien and Horvitz, 2000; Gumienny et al., 2001). CED-2/CED-5/CED-12 homologous signaling pathways in *Drosophila* and mouse were CG1587/myoblast city/Dmel, RKII/Dock180/ELMO1, and CED-1/CED-6 homologous signaling pathways were Drpr/dCed-6, MEGF10/GULP1 (Zheng et al., 2017), which are are highly conserved to regulate ACs clearance. Interaction between ABL-1 and ABI-1 inhibits ABI-1 and thus negatively regulates phagocytosis, but the study of this pathway in *Drosophila* and mammals remains to be determined (Hurwitz et al., 2009).

### 3.4 Degradation

The dynamics of PtdIns (4,5) P2 and PtdIns3P phosphatidylinositol is a very important event in the sealing of phagocytic vesicles (Flannagan et al., 2012; Cheng et al., 2015; Wang and Yang, 2016). PtdIns (4,5) P2, PtdIns3P are abundantly present in unconfined and confined phagosomes respectively. The model organism *C. elegans* has been relatively well studied in this regard. With the accumulation of PtdIns3P, the phagosome recruits SNX9 family protein LST-4 to the phagosome, and SNX9 further recruits DYN-1 to complete phagocytosis. Researches in *C. elegans*, *Drosophila* and mammalian cells show that abnormal Dyn-1 function results in stalled phagosome maturation and aggregation of apoptotic bodies within phagosomes, indicating that it plays a key role in phagosome maturation (Kinchen et al., 2008; Yu et al., 2008; Shklover et al., 2015).

The maturation of phagosomes undergo several processes, including early endosome, late phagosome and phagolysosome formation (Almendinger et al., 2011; Cheng et al., 2015). The different forms of membranous vesicles require a series of Rab GTPases proteins, which also participate in the acidification of phagocytic lysosomes. For example, the GTPases RAB-5 and RAB-7 bind to early and late endosomes respectively to mediate the processes (Li et al., 2009). It was shown that early phagosome recruits RAB-5 protein to assemble downstream factors while RAB-7 participates in the later stages of phagosome maturation and mediates phagocytosis and lysosomal fusion (Vieira et al., 2002; Kinchen and Ravichandran, 2008). In mammalian the Mon1 interacts with Rab5, and the Mon1-Ccz1 complex binds Rab7 and may affect Rab7 activation, so Mon1-Ccz1 may facilitate the transition from Rab5 to Rab7 through a Rab exchange mechanism, but whether SAND-1-CCZ-1 (Mon1-Ccz1) uses a similar mechanism to regulate phagosome formation in *S. hidradiata* remains to be determined (Kinchen and Ravichandran, 2010; Zheng et al., 2021).

Not only RAB-5, RAB-7 and LAMP-1 are recruited during the process of phagosome maturation, but also V-ATPase and other factors such as histone proteases. In nematode and zebrafish studies, V-ATPase was revealed to play a role in different stages of phagosome formation. In zebrafish V-ATPase is not only involved in the acidification of lysosomes, but may also play a role in phagosome maturation. In nematodes, it is required in the early stages of phagosome maturation (Zheng et al., 2021). After Rab7 recruitment to the phagosome, the HOPS complex begins to recruit, thereby activating Rab7 and facilitating its eventual fusion with the lysosomal structure (Kinchen and Ravichandran, 2008).

After phagocytic lysosome formation, the phagocytic lysosome recruits proteins to the surface and releases acid hydrolases, among others, to degrade ACs. In *C. elegans*, LAAT-1, the lysosomal lysine/arginine transporter maintains lysosomal persistence and releases lysosomal histone protease L (CPL-1) and DNase II(NUC-1) to control the digestion and degradation of ACs (Liu et al., 2012; Xu et al., 2014).

Finally, when apoptotic cells are degraded within the phagolysosomes, a large amount of metabolic cargo is produced, such as amino acids, lipids, nucleic acids and some other potentially cytotoxic macromolecules, so macrophages induce ABCA1 expression, allowing cholesterol efflux and reducing damage to membranes by harmful substances (Kiss et al., 2006; Han and Ravichandran, 2011). During the clearance of apoptotic cells, the vesicular cycle in the cell is also not negligible, and the process is regulated by the RAB family of GTPases. RAB17 is recruited into the phagosome containing apoptotic cells, thus mediating a vesicular cycle from the phagosome to the recycling endosome for the purpose of returning the cell surface area (Yin et al., 2019).

## 4 Transcription factor regulating apoptosis

In the past decades, we have made great progress in the study of apoptosis and ACs clearance, both in terms of mechanism and human diseases. However, in the large environment of the organism, there are multiple genes regulated by each other, protein-molecule interactions and signaling pathways to jointly maintain the homeostasis of the organism, so the research in this area still needs to be explored and exploited. As a hot topic of research in recent years, transcription factors play a crucial role in regulating the functions of organisms through the regulation of gene expression. Therefore, further study of apoptosis and apoptotic cell clearance by transcription factors has become an important direction to broaden the regulatory function of organisms.

Transcription factors are a class of proteins, interacting with cis-acting factors, which act as enhancers or silencers upstream of the transcription initiation region to enhance or inhibit gene expression respectively. The typical transcription factor contains three functional structural domains, namely the DNA binding domain, the transcriptional regulatory domain and the regulatory domain of other regulatory proteins, and individual transcription factors also have post-transcriptional regulatory domains (Ciarapica et al., 2003).

Generally, there are two categories of transcription factors classified by their action characteristics. The first category is general transcription factors, which can bind to RNA polymerase II to form transcription initiation complexes to enable downstream gene transcription and expression (Müller, 2001; Burley and Kamada, 2002). The second category of transcription factors is specific transcription factors, i.e., specific transcription factors required for individual gene expression. Transcription factors can be classified as zinc finger motif, helix-turn-helix (HTH), leucine zipper region (bZIP), homologous structural domains, nuclear receptors, etc. Based on the DNA binding domain, these categories account for more than 80% of human transcription factors (Vaquerizas et al., 2009; Lambert et al., 2018).

**FIGURE 1:**
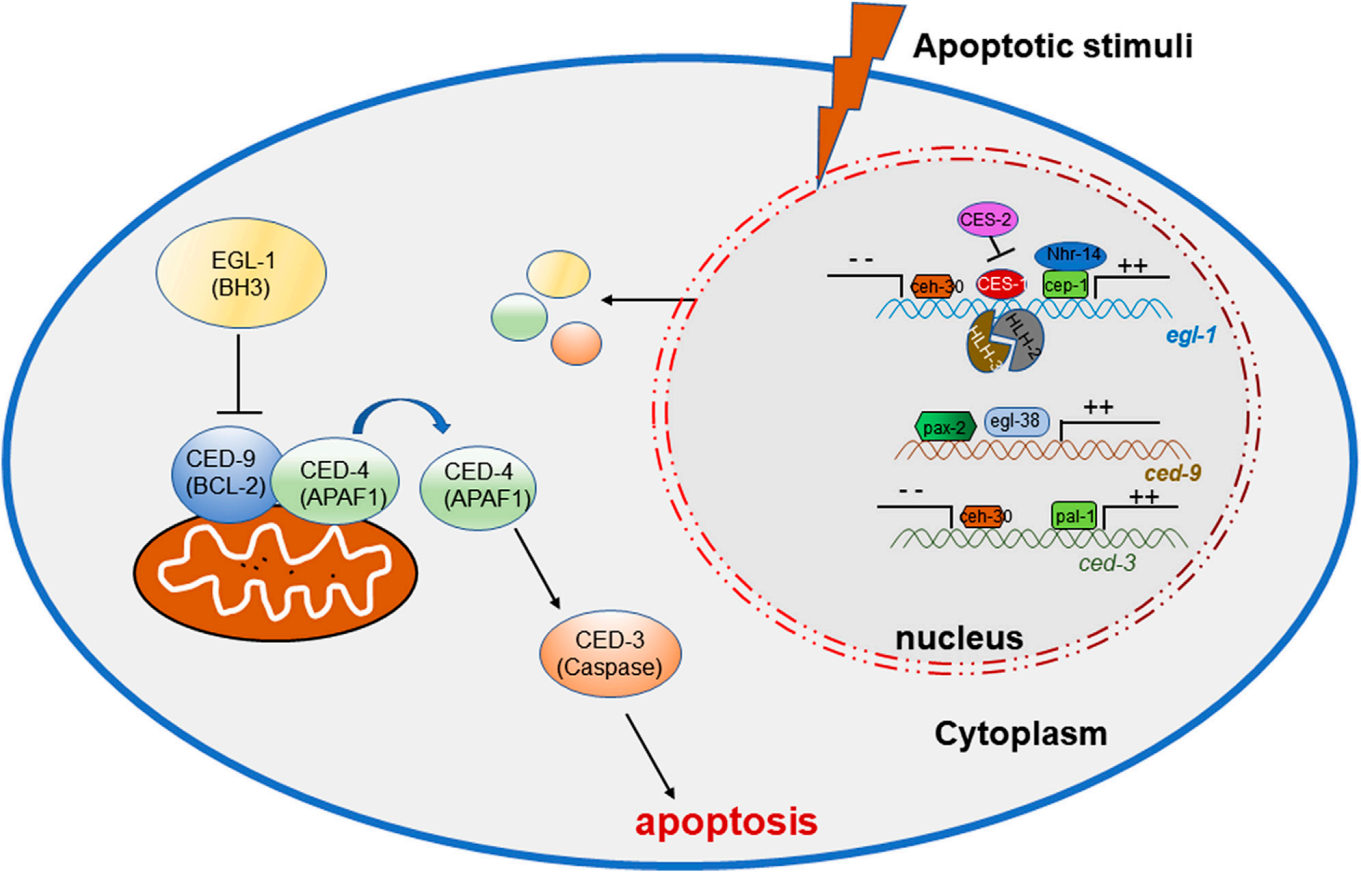
Transcription factors regulating the onset of apoptosis in *C. elegans*.

During the studies of apoptosis, researchers are seeking the roles of transcription factors and several key factors have been found to regulate the process. During the study of *C. elegans*, there are numerous correlations reporting the involvement of transcription factors in the apoptogenesis of ACs. Important factors in apoptosis, such as *ced-3*, *ced-4*, *ced-9* and *egl-1*, are activated or suppressed by multiple transcription factors (Figure 1) (Wang and Yang, 2016), to initiate the normal occurrence of apoptosis, promoting the normal development and maintaining the homeostasis of the organism (Table 1).

**TABLE 1:** The homologs of transcription factors regulating apoptosis in *C. elegans*.

Transcription Factor	Regulatory target	Function for apoptosis	Human homologue
CEP-1	*egl-1*	Promote	P53
CES-1		Inhibit	SLUG
CES-2		Promote	HLF
HLH-2		Promote	HLH
HLH-3		Promote	HLH
CEH-30		Inhibit	BARHL2
EGL-38	*ced-9*	Promote	PAX
PAX-2		Promote	PAX
PAL-1	*ced-3*	Promote	CDX2

CDX, caudal-type homeobox transcription factor; HLF, hepatic leukaemia factor; Pax, paired box gene; HLH, helix-loop-helix transcription factor; SLUG, a zinc finger protein and Snail family member; P53, tumor suppressor gene; BARHLL2, the Bar homeodomain transcription factor.

In *C. elegans*, the p53 homolog CEP-1 is required for DNA damage-induced germ cell death by directly regulating the *egl-1* transcripts (Hofmann et al., 2002), and NHR-14, which belongs to nuclear hormone receptors (NHRs), regulates *egl-1* cooperating with CEP-1 to mediate germ cell apoptosis (Sang et al., 2022). During development, CES-1 inhibits the transcriptional expression of *egl-1* through binding the Snail-binding sites in the *elg-1* promoter, thus blocks programmed cell death in specific neurons. In *C. elegans*, ces-1 encodes a C2H2-type zinc finger transcription factor which belongs to the Snail family proteins, which transcription is negatively regulated by the bZIP transcription factor CES-2 by binding CES-1 upstream sequences *in vitro* and thus may directly repress *ces-1* transcription *in vivo* (Metzstein and Horvitz, 1999). In regulating EGL-1 expression, the bHLH transcription factors HLH-2 and HLH-3 forms a heterodimer to bind to Snail-binding sites of the *egl-1* locus *in vitro* and regulates the death of the NSM sister cells (Thellmann et al., 2003). *ceh-30* is a Bar homeodomain transcription factor, encoding a homeodomain protein most similar to *Drosophila* and mammalian BarH1. CEH-30 blocks the death of the four male-specific cephalic companion neurons (CEMs) by repressing the transcription of both the *egl-1* and *ced-3* genes (Nehme et al., 2010).

The *C. elegans* gene *egl-38* and *pax-2* encodes a Pax transcription factor that is most similar to the mammalian Pax2/5/8 subclass of factors (Chamberlin et al., 1997), which have been proved to influence cell death and promote cell survival. Work in *C. elegans* shows that egl-38 and pax-2 act as a positive transcriptional regulators of *ced-9* by directly binding to regulatory sequences upstream, thus influence both somatic and germline cell death (Park et al., 2006).

PAL-1, the *C. elegans* homolog of the mammalian tumor suppressor gene Cdx2, can bind to the *ced-3* promoter sites and directly activate *ced-3* transcription (Maurer et al., 2007), in order to control *ced-3* expression and cell death in worm tail-spike cells.

Meanwhile, previous reports in mammals demonstrate that transcription factors promote normal cell reproduction and physiological activities by regulating various genes expression. For example, in response to different apoptotic stimuli, different genes are activated through transcription factors binding to specific DNA sequences, in order to promote or repress their expression. DNA damage gives rise to the production of the tumor supressor gene p53 (Gottlieb and Oren, 1998; Yu and Zhang, 2005), which activates the transcription of many pro-apoptotic genes and restain expression of oncogenes, such as c-Myc and E2F1, ultimitely leads to apoptosis (Thompson, 1998; Stanelle and Pützer, 2006). Pro-inflammatory cytokines or growth factors through NF-κB, IRF, STAT (signal transducer and activator of transcription) or FOXO family transcription factors are also proved to involve in apoptosis (de Martin et al., 1999; Fu, 1999; Zhang et al., 2011); transcription factors involved in apoptosis such as STAT92E, p53 and NF-κB have also been found in *Drosophila* (Betz et al., 2008; Tavignot et al., 2017; Zhou, 2019). Therefore, the study of transcription factors in apopptosis is of great importance in immune regulation, body homeostasis, and human disease treatment.

## 5 Transcriptional regulation of apoptotic cells clearance

Transcription factors are also involved in the clearance process of ACs. The current research on transcription factors is mainly focused on the process of macrophage maturity and activation, but the other processes are less involved at present.

### 5.1 Transcription factors regulate “find me” signaling

During efferocytosis, ACs can attract phagocytes by releasing “find me” signals, such as nucleotides, chemokines and their modified membranes. These molecules can stimulate the migration of macrophages to ACs, but the recruitment of find me signals to macrophages depends on many other factors, such as the type of phagocytes and ACs and the stimulation of apoptosis (Ravichandran, 2010).

S1P, a type of lysophospholipid, is a sphingosine metabolite produced by sphingosine kinase (SphK, SphK1/SphK2) acting on sphingosine as an important “find me” signal to recruit phagocytes (Gude et al., 2008; Luo et al., 2016). S1PR, the receptor for S1P, which belongs to the G protein-coupled receptor family, is required for cell survival, cell migration, apoptosis, and inflammation through binding to S1P. During apoptosis induced by DNA damage, p53 accumulates which in turn activates the lysosomal pathway as well as the mitochondrial pathway. The mitochondrial pathway causes caspase enzyme activation and the lysosomal pathway releases cathepsins into the cytoplasm. The proteases released by both pathways cause downregulation of SphK1, which in turn reduces intracellular S1P levels (Taha et al., 2004). However, it is not clear whether the regulation of SphK1 by proteases occurs through direct cleavage or some other indirect mechanism. Binding of extracellular S1P to S1PR reduces the production of pro-inflammatory factors and anti-inflammatory factors (e.g., IL-10), vascular endothelial growth factor (VEGF), nuclear transcription factor peroxisome proliferator-activated receptor *λ* (PPARλ), and erythropoietin EPO is also upregulated, thereby stimulating the anti-inflammatory macrophage phenotype, promoting the polarization of macrophage M2, and enhancing efferocytosis (Cheng et al., 2015). S1P also inhibits macrophage death, induces COX-2 expression, promotes cAMP production, and inhibites NF-κB signaling. In addition, binding of extracellular S1P to S1PR1 promoted phagocytic vesicle maturation after pathogen uptake (Weigert et al., 2009).

**FIGURE 2:**
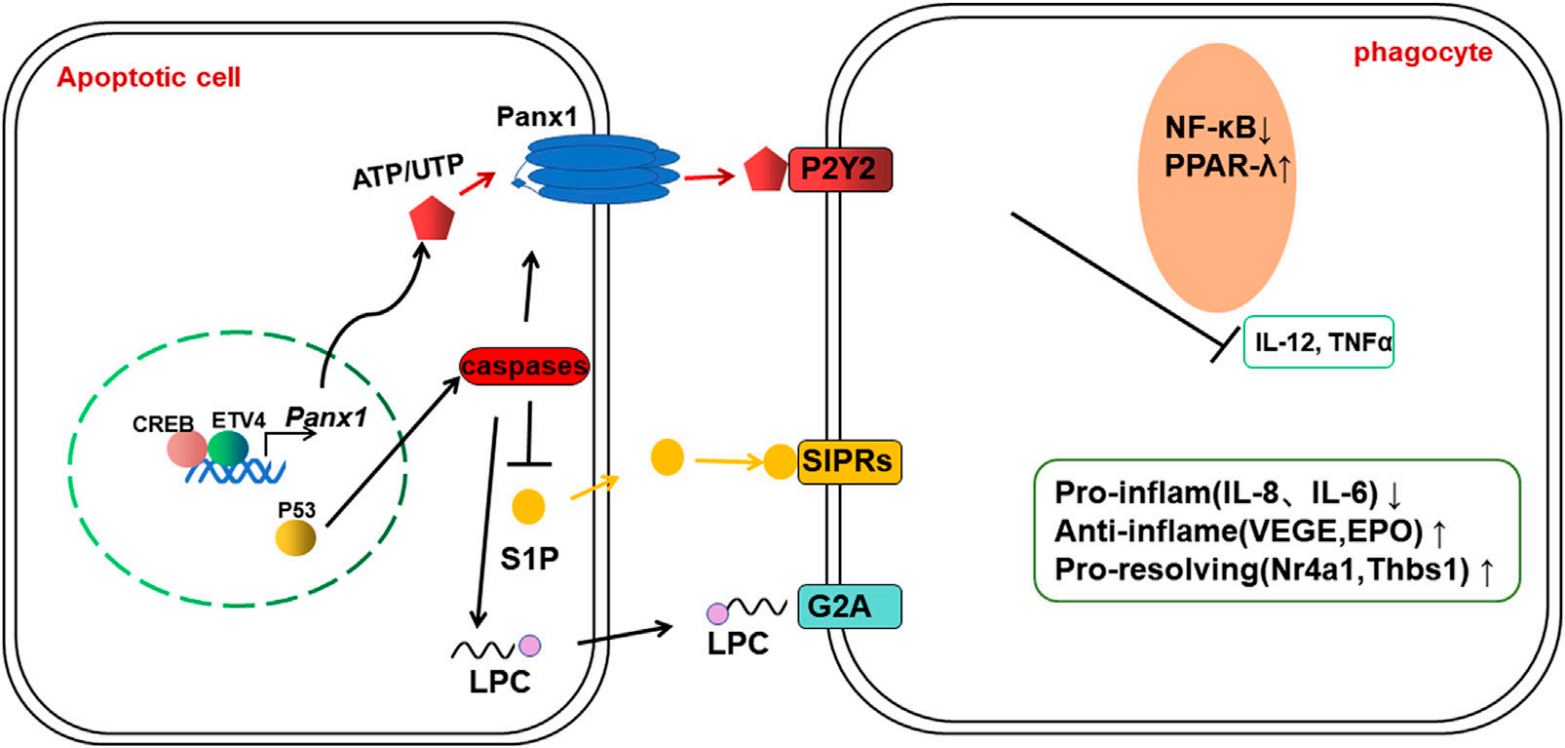
Transcription factors regulating the release and recognition of “find me” signals.

Nucleotides can also be used as find me signals to recruit phagocytes (Elliott et al., 2009). Nucleotides are released extracellularly in a time- and caspase-dependent manner at low levels of ATP as well as UTP. In ACs, the plasma membrane channel Pannexin-1 (PANX1) mediates the release of ATP and UTP by forming hexameric channels in a caspase-dependent manner, promoting the releasing of nucleotides, thus mediates the recruitment of macrophages by binding to the nucleotide receptor P2Y2 (Chekeni et al., 2010). Previous study showed the expression of *Panx1* was initiated and activated by transcription factors CREB and ETV4 in the rat epididymis (Dufresne and Cyr, 2014), which may give us an inspiration on the regulatory pattern of find me signals. The conversion of extracellular ATP into adenosine, which is the result of the interaction between CD39 and CD73 (Idzko et al., 2014). Adenosine binds to the adenosine A2A receptor on the surface of macrophages and subsequently inhibits NF-κB signaling and upregulates the expression of Thbs1 and the nuclear receptor gene Nr4a. Thbs1 is a major activator of TGFβ, while members of the Nr4a family restrain the level of pro-inflammatory cytokines such as TNFα and IL-8 in macrophages and promote the efferocytosis process (Elliott et al., 1950; Yamaguchi et al., 2014) (Figure 2).

Since the research on find me signaling is relatively limited, the specific release mechanism of “find me” signaling during recruitment and how transcription factors regulate the process of recruiting macrophages remain to be discovered, which means that this area is well worth investigating.

### 5.2 Transcription factors regulate “eat-me” signaling

The migration and proximity of phagocytes to ACs depends on find me signals, while the specific recognition and binding to phagocytes depends on the exposure of eat me signals. The most studied and well-known eat me signal- PS, exposed on the cell surface of ACs by the co-regulation of phospholipid scramblase and flippase depending on the caspases activity (Segawa and Nagata, 2015).

Phagocytes recognize and bind these signals *via* their own PS recognition membrane receptors directly or indirectly *via* bridging molecules, thereby promote the clearance of ACs and prevent inflammation. Over the past few years, scientists realized that various receptors on phagocytes can recognize PS exposed on ACs. Tim4 expressed in lymphocytes of various mouse tissues, was found to bound and engulf ACs through recognizing PS by its immunoglobulin domain (Miyanishi et al., 2007). BAI1, which belongs to the adhesion-type G-protein-coupled receptor family, was reported as a PS recognition receptor, which formed a trimeric complex with ELMO and Dock180 to facilitate engulfment of ACs (Park et al., 2007). Stab2 which expresses in human monocyte-derived macrophages, mediates the clerance of aged red blood cells and ACs by recognizing PS (Park et al., 2008). MFG-E8 can simultaneously recruit integrin α_v_β_3_ on phagocytes and recognize PS to uptake thre ACs. Gas6 and protein S, are involved in concatenate PS exposed on ACs to TAM receptors on phagocytes as bridging molecules (Anderson et al., 2003; Hanayama et al., 2004; Toda et al., 2012).

The expression of these receptors, and bridging molecules, enhances phagocyte recognition of ACs. Transcription factors such as the nuclear receptor super-family, STAT family and NF-κB have been found to be involved in the regulation of PSR, complement molecules and other eat me signals, which are important for the regulation of inflammation, the efficient and timely elimination of ACs and the stability of body’s immune system.

The peroxisome proliferator-activated receptor (PPAR) and the liver x receptor (LXR), which belong to the nuclear receptor superfamily, are involved in cellular lipid homeostasis. PPAR (heterodimers *α*, *β*/*δ* and *γ*) and LXR (heterodimers *α*, *β*) are ligand-activated transcriptional activators with different tissue expression. After binding to ligands such as fatty acids and oxysterols, PPAR and LXR then form heterodimers with the retinoid X receptor (RXR) and recruit co-activators to induce transcription of various genes involved in lipid and cholesterol metabolism (Han and Ravichandran, 2011). During ACs clearance, LXR and PPAR activate form heterodimers with RXR and upregulate phagocytic receptors and regulators. Existing studies have shown that activation of LXR and PPAR during apoptotic cell clearance leads to upregulation of phagocytic receptors (e.g.,: Mer) as well as of modulators (C1qb,Gas6,MFG-E8d) (A-Gonzalez et al., 2009; Mukundan et al., 2009). Here the clearance of apoptotic cells is influenced by two main pathways. In the first pathway, phagocytosis of apoptotic cells leads to the activation of LXR as well as PPAR, which upon activation regulates the expression of relevant phagocytic receptors as well as regulators. In the second pathway, recognition and binding of phagocytic receptors to phosphatidylserine activates LXR and PPAR, which in turn promotes the expression of Abca1, which not only induces cholesterol efflux but also promotes efferocytosis (Hamon et al., 2002). However, it is still unclear what ligands LXR and PPAR bind, how LXR and PPAR receive signals delivered by phosphatidylserine, and how they are transformed into macrophage lipid metabolism. Deficiency of RXR affects the transcription of cell surface receptors (e.g., CD36, Fcgr1, MERTK, Axl, etc.), regulators (e.g., C1qa, C1qb, C1qc, etc.) and transglutaminase-2 (Tgm2), which play important roles in cytokinesis and other macrophage functions.

PPAR-δ, PPAR-γ and RXRα have been demonstrated to upregulate MERTK and AXL transcriptional levels in macrophages (Han and Ravichandran, 2011). Macrophages lacking PPARγ, PPARβ/δ or RXRα (Vaquerizas et al., 2009; Gutiérrez-González et al., 2019), or PPARγ (Naeini et al., 2020) affect Axl transcription and inhibit ACs uptake (Röszer, 2017), causing macrophage adhesion and migration to be blocked (Garabuczi et al., 2015; Röszer, 2017). During efferocytosis, Axl and Mer signaling pathways directly inhibit Toll-like receptor (TLR) and type I IFN-driven inflammatory signaling pathways by different mechanisms. In dendritic cells, activation of Mer by ACs inhibits the IκB kinase IKK activity downstream of TLR4, suppressing NF-κB and reducing its binding to the TNF promoter. In addition, the reduction of TNFα is mediated by activation of Axl receptor tyrosine kinase and inducing of Twist transcriptional repressor, which binds to the E box region of the TNF promoter and inhibits NF-κB-dependent transcription (Sen et al., 2007).

**FIGURE 3:**
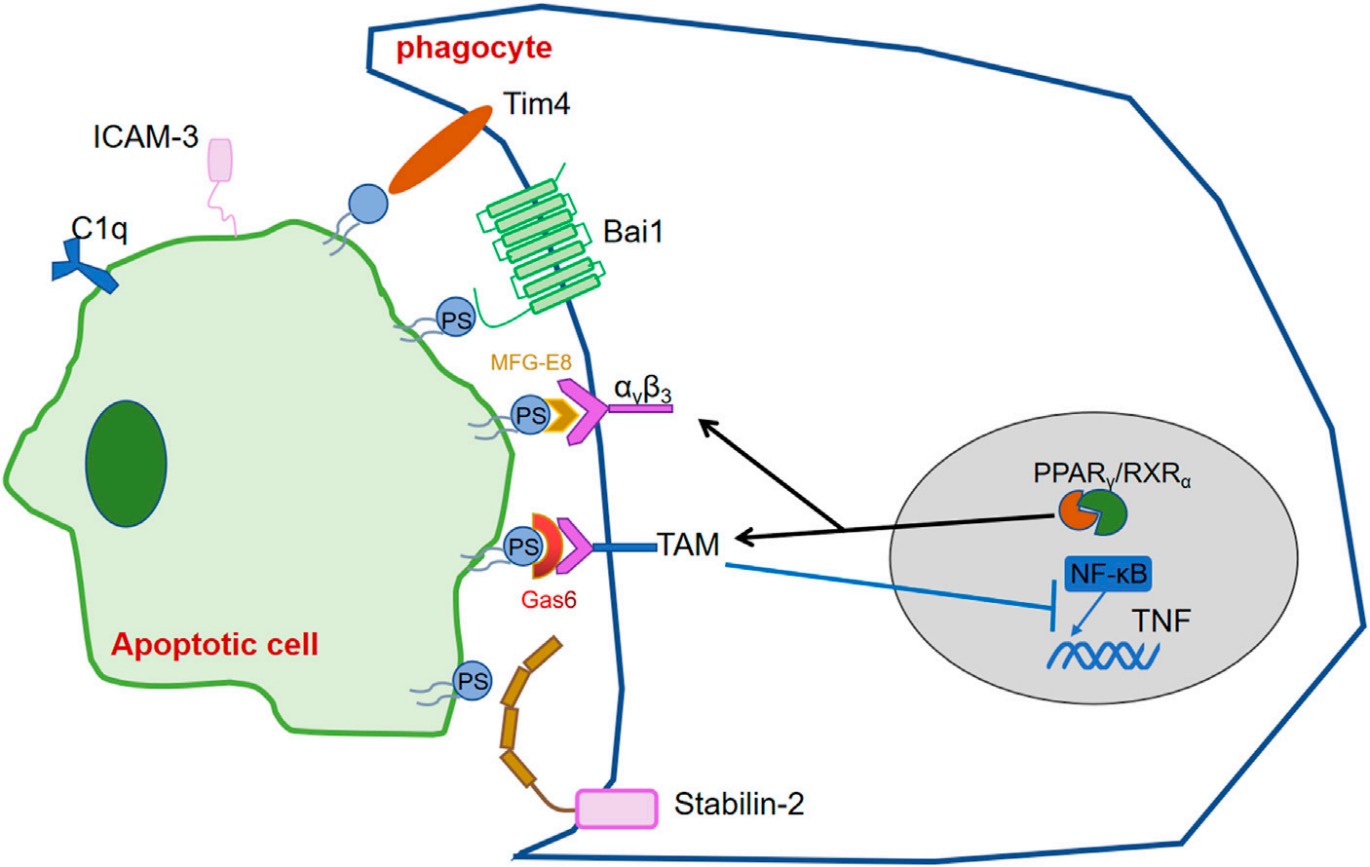
Transcription factors regulating the release and recognition of “eat me” signals.

MERTK is indirectly controlled by the glucocorticoid GC, which has been shown to upregulate LXR/RXR expression and eventually increase the uptake of ACs. Glucocorticoids increase the phagocytic ability to ACs both in short-term and subsequent phagocytosis. Short-term phagocytosis is mainly elevated by increasing of MERTK and C1q expression levels, whereas sustained phagocytosis acts by promoting the expression of LXR, PPARδ and UCP2 (Garabuczi et al., 2015) (Figure 3).

### 5.3 Transcription factors regulate apoptotic cell clearance and degradation

**FIGURE 4:**
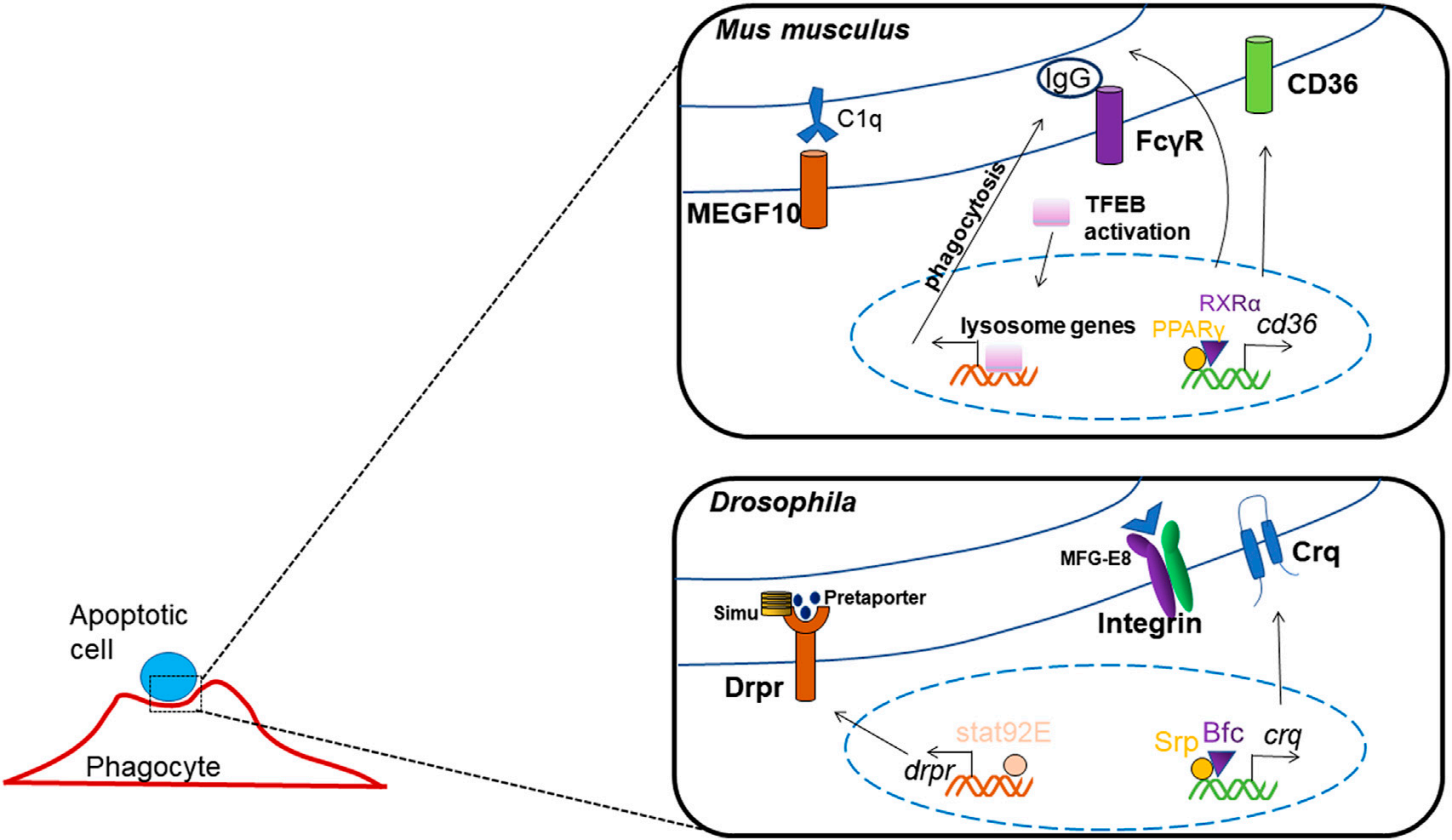
Transcription factors regulating apoptotic cell clearance.

Macrophages internalize pathogens or ACs through enveloping them into vesicles called phagosomes, which then fuse with lysosomes to mature into phagolysosomes and ultimately degrade the pathogens or ACs. The cellular mechanism has been revealed relatively clear and detailed, yet the roles of transcription factors in controlling apoptotic cell clearance are less reported (Figure 4). The most studied transcription factors, which regulate apoptotic cell clearance are PPARs, LXR, RAR, RXR and GR.

Researchers have found that the activation of Fcγ-receptor which mediated phagocytosis and endocytosis, causes nuclear transposition of TFEB, which enhances the expression of lysosome genes, TFEB silencing reduces the enhancements in degradation and bacterial killing mediated by Fcγ-receptor (Gray et al., 2016).

The expression of the class B scavenger receptor *CD36* was regulated by transcription factors PPARγ and RXRα, as the *Cd36* promoter includes a interaction element for PPARγ/RXRα heterodimers, thus activates the process of pathogen elimination and apoptotic cell recognition (Silverstein and Febbraio, 2009; Röszer, 2017). And, the homolog of CD36 in *Drosophila*, *crq* was found to be transcriptional regulated by GATA factor and its co-factor, Bfc, which interacts with Srp zinc finger domain to strengthen this binding; thus, they function together in boosting *crq* expression and efferocytosis (Zheng et al., 2021). In *Drosophila*, it has also been shown that Srp may regulate the expression of factors mediating phagosome maturation and apoptotic cell degradation, thus the process of ACs clearance may be influenced by its deletion (Shlyakhover et al., 2018).

Another phagocytotic receptor in *Drosophia*, Drpr, has been reported to be regulated by transcription factor Stat92E, which can directly binds to the promoter of *drpr*, and mediates glial phagocytosis to axonal debris (Doherty et al., 2014).

In studies of TIM-1-mediated acute kidney injury, binding of TIM-1 to ACs triggers TIM-1 phosphorylation and the recruitment of p85, which interact with each other to block TLR4 expression, or the phosphorylation and activation of NF-κB, resulting in an anti-inflammatory phenotype and phagocytosis (Yang et al., 2015).

Immunoglobulins G and M (IgG, IgM) and complement binding to ACs provide eat me signals for macrophages. Increased PPARγ and RXR ligands promote IgG or IgM recognition by ACs, while the absence of PPARγ or RXRα reduces the expression of complement factors (e.g., C1q), thus inhibits the binding and uptake of ACs by macrophages (Roszer et al., 1950; Mukundan et al., 2009).

## 6 Conclusion

In the last few decades, we have made great progress in the study of apoptotic cell clearance. In living organisms, molecules, proteins and signalling pathways interact with each other, and the signalling pathways and functions involved are complex and diverse. However, there are still many regulators involved in apoptotic cell clearance waiting to be discovered and clarified. At the same time, the presence of transcription factors, a hot topic of research in recent years, regulates gene expression and affects the function of the organism.

The regulatory role of transcription factors is not entirely point-to-point; it may be a one-to-many or many-to-one process, hence the complexity of its study. In previous studies, numerous transcription factors such as the nuclear receptor superfamily, IRF, AP-1, NF-κB and STAT family have been identified as regulating the clearance of ACs. Through the regulation of genes participating in the clearance of ACs, transcription factors directly or indirectly influence the recognition of ACs, the maturation of macrophages and the degradation of ACs, leading to the development of organs and maintenance of the immune system. As impaired clearance of apoptotic cells leads to human diseases, the NR superfamily and transcription factors such as AhR have been documented as important targets for the prevention and treatment of hyperlipidaemia, diabetes and chronic inflammatory diseases including atherosclerosis, as well as autoimmune diseases.

In conclusion, transcription factors play important roles in the regulation of ACs clearance. There are still some unexplored signaling pathways between ACs and macrophages, and the current articles on the involvement of transcription factors in the process of apoptotic cell clearance are relatively superficial, and there are still many questions waiting to be explored, such as how transcription factors regulate how transcription factors regulate the shutdown of efferocytosis when apoptotic cell clearance is completed and how the high load/continuous b efferocytosis is transcriptionally regulated, etc. Therefore, finding new signaling pathways through transcription factors would be a valuable approach to broaden the field of apoptotic cell clearance. At the same time, with the advances in big data analysis and experimental techniques, it is hopeful that researchers will be able to broaden the field of research while working together to provide new insights and avenues to treat human diseases. There is an intriguing, but still not fully understood transcriptional mechanism between ACs and macrophages, which requires for further studies in the revelation of transcriptional regulation in ACs clearance and its therapeutic use.

## Glossary

PCD: Programmed cell death
TNF: tumor necrosis factor
M1: classically activated macrophages
M2: alternative activated macrophages
LPC: lysophosphatidylcholine
S1P: sphingosine 1-phosphate
ICAM3: Intercellular adhesionmolecule 3
PS: phosphatidylserine
PSR: PS recognizing membrane receptor
BAI1/3: Brain Angiogenesis Inhibitor 1/3
TIM4: T cell immunoglobulin mucin receptor 4
Stab2: Stabilin-1/2
TLT2: (TREM)-like protein 2
TAM: receptor tyrosine kinase Mer Tyro3 and Axl
Gas6: growth arrest-specific gene 6
Drpr: Draper
Crq: Croquemort
HTH: helix-turn-helix
bZIP: leucine zipper region
STAT: signal transducer and activator of transcription
SphK: sphingosine kinase
VEGF: vascular endothelial growth factor
PPARλ: peroxisome proliferator-activated receptor λ
PANX1: Pannexin-1
LXR: liver x receptor
RXR: retinoid X receptor
Tgm2: transglutaminase-2
TLR: Toll-like receptor
AP-1: activator protein 1
IRF: interferon regulatory factor
SOCS: suppressors of cytokine signaling
IFNAR: type Ⅰ interferon receptor
IFNBR: type II interferon receptor
CSF: colony-stimulating factors
JAK: Janus kinase
RAR: retinoid receptor
GR: glucocorticoid receptor
RR: retinoid-like receptors
ATRA: all-trans retinoic acid
CRA: cis-retinoic acid
iNOS: inducible nitric oxide synthase
AhR: Aryl hydrocarbon receptor
IgG, IgM: Immunoglobulins G and M
